# Distance and Psychoanalysis: Negotiating with the Virtual World

**DOI:** 10.1192/j.eurpsy.2023.1718

**Published:** 2023-07-19

**Authors:** V. Gairola

**Affiliations:** Department of Liberal Arts, Indian Institute of Technology, Hyderabad, Noida, India

## Abstract

**Introduction:**

Earlier ways of being in touch with each other have been turned into nostalgia the way COVID-19 did and continues to shake the world. There has been an enormous move under COVID-19 to move towards zoom, telephones, etc. to do online psychoanalysis and psychotherapy. Audio and video have taken exceptional agential forms by replacing physical hearing and seeing. Physical touch is replaced by a virtual touch. The virtual has extended the meaning of the body, feelings, sensations, and relations.

**Objectives:**

The aim of this paper lies in understanding, demystifying, and de-alienating the relationship between distance and psychoanalysis. It is to understand what ‘virtual turn’ entails in therapy. This paper theorizes the ‘and’ between distance and psychoanalysis.

**Methods:**

This research used primary sources like books and articles to elucidate the possibilities and challenges of distance therapy.

**Results:**

In-office analysis, analysis with the video, and telephone analysis bring their own unique ways of communication and understanding. “Talking cure” and “chimney sweeping” come closest to the domain of telephone analysis where voice again becomes the foreground. Technology and distance therapy’s relation to the analytic position is understood critically as what they mean for both the therapist and the patient in such times of shared social crisis.

**Conclusions:**

International Psychoanalytic Association (IPA) has authorized telephone and virtual analysis, which is a commendable step as this makes analysis far more accessible than it has ever been to people who live outside major cities along with breaking the hierarchy between the patient and the analyst.

**Disclosure of Interest:**

None Declared

